# Supplementary material for: PIK3CA Gene Mutations in HNSCC: Systematic Review and Correlations with HPV Status and Patient Survival
Source: Cancers (Basel). 2022 Mar 2;14(5):1286. doi: 10.3390/cancers14051286 (PMC8909011; doi:10.3390/cancers14051286)
Supplement: Supplementary file 1 [file cancers-14-01286-s001.zip › Figure S3-Flow chart from Sistematic-review Inclusion criteria.pdf]

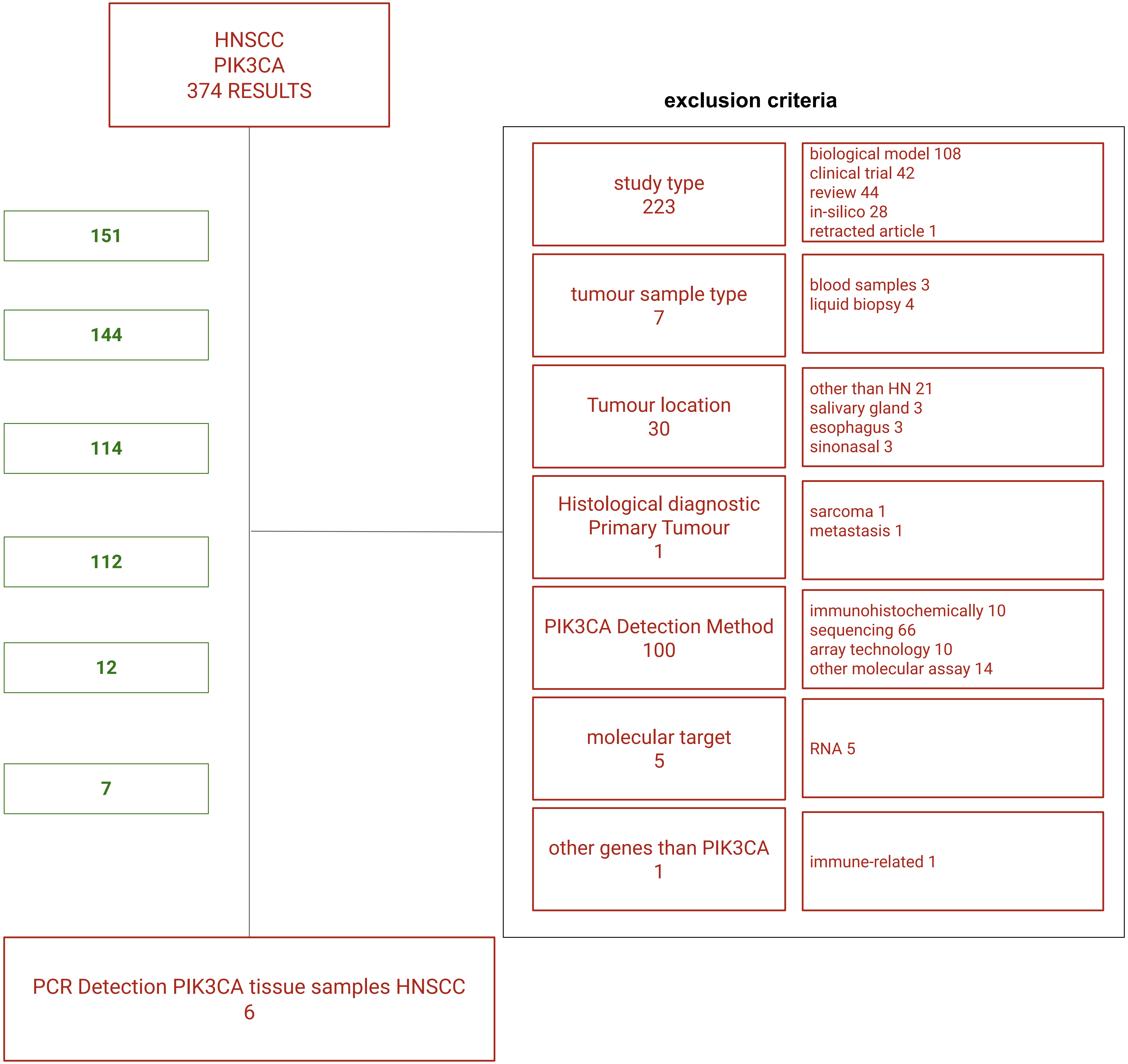
HNSCC
PIK3CA
374 RESULTS
exclusion criteria
151
144
114
112
12
7
study type
223
biological model 108
clinical trial 42
review 44
in-silico 28
retracted article 1
tumour sample type
7
blood samples 3
liquid biopsy 4
Tumour location
30
other than HN 21
salivary gland 3
esophagus 3
sinonasal 3
Histological diagnostic
Primary Tumour
1
sarcoma 1
metastasis 1
PIK3CA Detection Method
100
immunohistochemically 10
sequencing 66
array technology 10
other molecular assay 14
molecular target
5
RNA 5
other genes than PIK3CA
1
immune-related 1
PCR Detection PIK3CA tissue samples HNSCC
6

working excluded data: sequencing

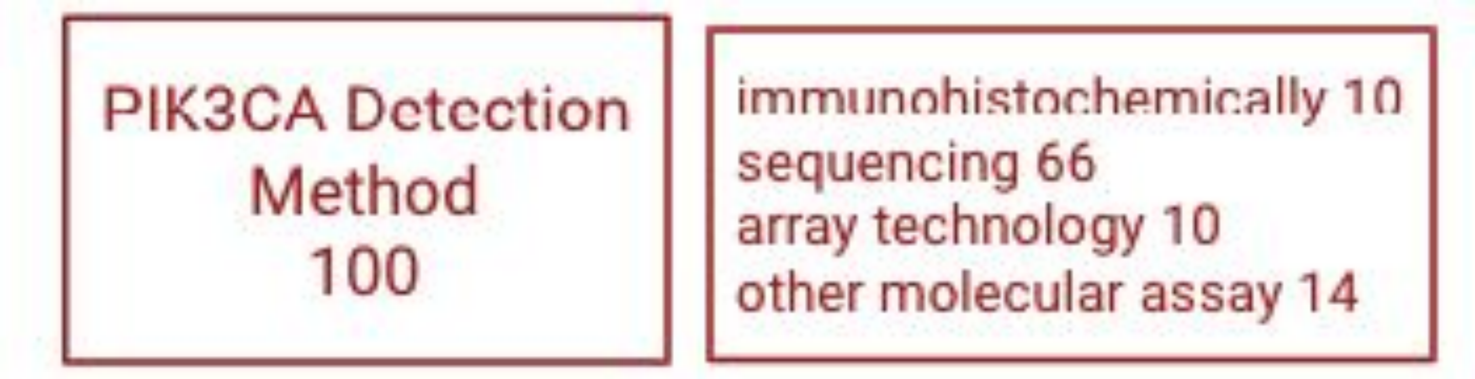

<u>HNSCC PIK3CA Mutation profile</u>: 66 articles eligible

Exclusion Criteria:

- molecular Target RNA
- without PIK3CA mutation profile data
- no more than 5 patient cases

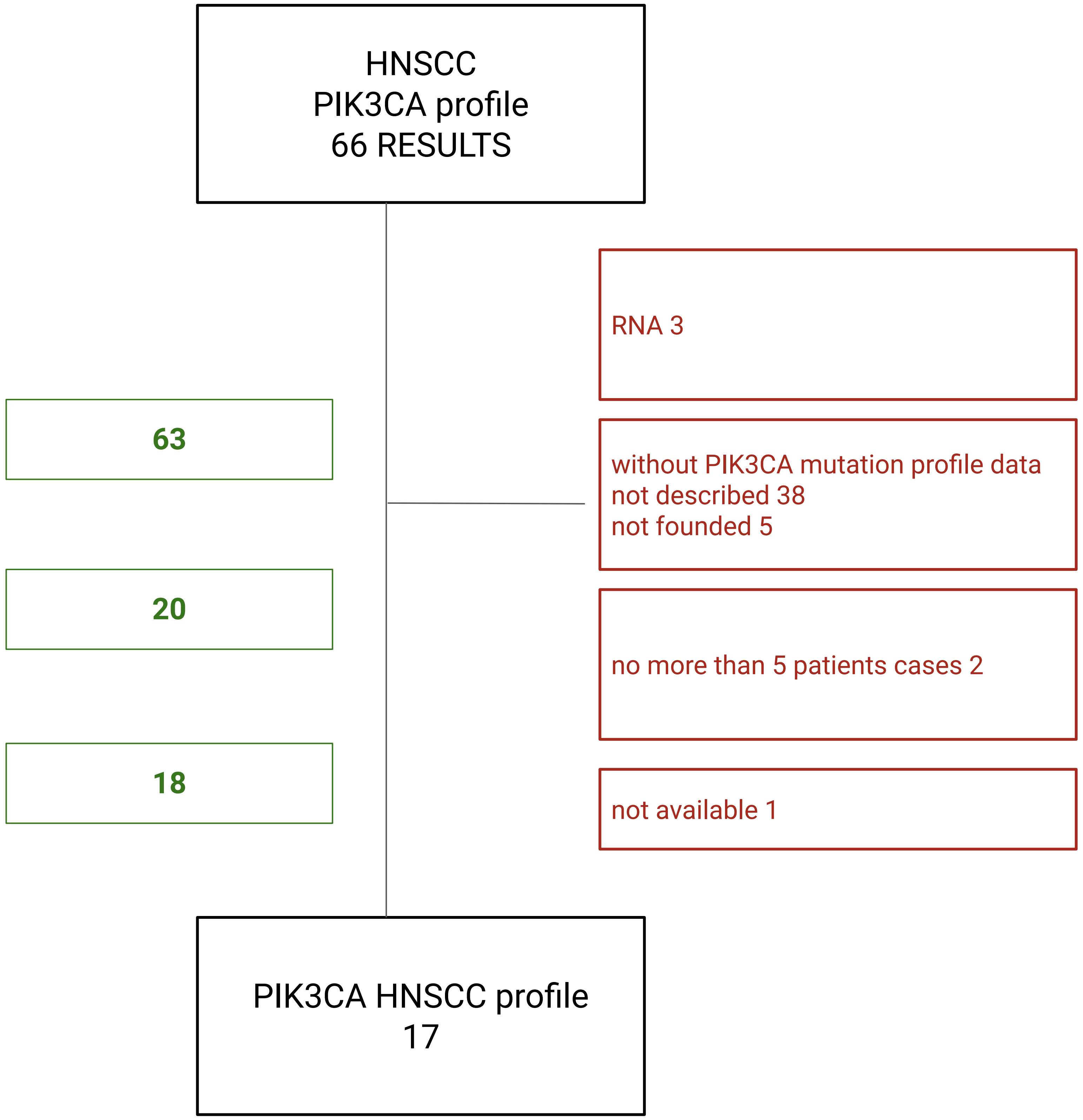

[1] Weiping Jie, et al 2021
[2] Alsofyani AA, et al 2020
[3] V. Denninghoff, et al 2020
[4] Ghias K, et al 2020
[5] Haft S, et al 2019
[6] Lim SM, et al 2019
[7] García-Carracedo D, et al 2016
[8] Al-Amri AM, et al 2016
[9] Chau NG, et al 2016
[10] Shah S, et al 2015
[11] Chen SJ, et al 2015.
[12] Cortelazzi B, et al 2015
[13] Nichols AC, et al 2013
[14] Cohen Y, et al 2013
[15] Bruckman KC, et al 2010
[16] Qiu W, et al 2008
[17] Qiu W, et al 2006
