# Supplementary material for: PIK3CA Gene Mutations in HNSCC: Systematic Review and Correlations with HPV Status and Patient Survival
Source: Cancers (Basel). 2022 Mar 2;14(5):1286. doi: 10.3390/cancers14051286 (PMC8909011; doi:10.3390/cancers14051286)
Supplement: Supplementary file 1 [file cancers-14-01286-s001.zip › Supplement_Systematic review References.pdf]

Supplement Systematic Review References

Supplemental Table S2

[1]Borkowska, Edyta M., et al. “Detection of Pik3ca Gene Mutation in Head and Neck Squamous Cell Carcinoma Using Droplet Digital Pcr and Rt-Qpcr.” *Biomolecules*, vol. 11, no. 6, May 2021, doi:10.3390/biom11060818.

[2]García-Escudero, Ramón, et al. “Overexpression of PIK3CA in Head and Neck Squamous Cell Carcinoma Is Associated with Poor Outcome and Activation of the YAP Pathway.” *Oral Oncology*, vol. 79, no. February, Elsevier, 2018, pp. 55–63, doi:10.1016/j.oraloncology.2018.02.014.

[3]Pattle, Samuel B., et al. “Copy Number Gain of 11q13.3 Genes Associates with Pathological Stage in Hypopharyngeal Squamous Cell Carcinoma.” *Genes, Chromosomes and Cancer*, vol. 56, no. 3, Mar. 2017, pp. 185–98, doi:10.1002/gcc.22425.

[4]Theurer, Julie A., et al. “Feasibility of Targeting PIK3CA Mutations in Head and Neck Squamous Cell Carcinoma.” *Pathology and Oncology Research*, vol. 22, no. 1, Jan. 2016, pp. 35–40, doi:10.1007/s12253-015-9970-3.

[5]McBride, Sean M., et al. “Mutation Frequency in 15 Common Cancer Genes in High-Risk Head and Neck Squamous Cell Carcinoma.” *Head & Neck*, vol. 36, no. 8, Aug. 2014, pp. 1181–88, doi:10.1002/hed.23430.

[6]Suda, Toshihito, et al. “Copy Number Amplification of the PIK3CA Gene Is Associated with Poor Prognosis in Non-Lymph Node Metastatic Head and Neck Squamous Cell Carcinoma.” *BMC Cancer*, vol. 12, BMC Cancer, Sept. 2012, doi:10.1186/1471-2407-12-416.

Supplemental Table S3

[1]Jie, Weiping, et al. “Multi-Site Tumour Sampling Improves the Detection of Intra-Tumour Heterogeneity in Oral and Oropharyngeal Squamous Cell Carcinoma.” Frontiers in Medicine, vol. 8, May 2021, p. 670305, doi:10.3389/fmed.2021.670305.

[2]Alsofyani, Abeer A., et al. “Molecular Characterisation in Tongue Squamous Cell Carcinoma Reveals Key Variants Potentially Linked to Clinical Outcomes.” Cancer Biomarkers, vol. 28, no. 2, May 2020, pp. 213–20, doi:10.3233/CBM-190897.

[3]Denninghoff, V., et al. “Mutational Status of PIK3ca Oncogene in Oral Cancer—In the New Age of PI3K Inhibitors.” Pathology - Research and Practice, vol. 216, no. 1, 2020, p. 152777, doi:https://doi.org/10.1016/j.prp.2019.152777.

[4]Ghias, Kulsoom, et al. “Mutational Landscape of Head and Neck Squamous Cell Carcinomas in a South Asian Population.” Genetics and Molecular Biology, vol. 42, no. 3, Sept. 2019, pp. 526–42, doi:10.1590/1678-4685-gmb-2018-0005.

[5]Haft, Sunny, et al. “Mutation of Chromatin Regulators and Focal Hotspot Alterations Characterize Human Papillomavirus–Positive Oropharyngeal Squamous Cell Carcinoma.” Cancer, vol. 125, no. 14, July 2019, pp. 2423–34, doi:10.1002/cncr.32068.

[6]Lim, Sun Min, et al. “Investigating the Feasibility of Targeted Next-Generation Sequencing to Guide the Treatment of Head and Neck Squamous Cell Carcinoma.” Cancer Research and Treatment, vol. 51, no. 1, Jan. 2019, pp. 300–12, doi:10.4143/crt.2018.012.

[7]García-Carracedo, Darío, et al. “Impact of PI3K/AKT/MTOR Pathway Activation on the Prognosis of Patients with Head and Neck Squamous Cell Carcinomas.” Oncotarget, vol. 7, no. 20, May 2016, pp. 29780–93, doi:10.18632/oncotarget.8957.

[8]Al-Amri, Ali M., et al. “Novel Mutations of PIK3CA Gene in Head and Neck Squamous Cell Carcinoma.” Cancer Biomarkers, vol. 16, no. 3, Mar. 2016, pp. 377–83, doi:10.3233/CBM-160576.

[9]Chau, Nicole G., et al. “Incorporation of Next-Generation Sequencing into Routine Clinical Care to Direct Treatment of Head and Neck Squamous Cell Carcinoma.” Clinical Cancer Research, vol. 22, no. 12, June 2016, pp. 2939–49, doi:10.1158/1078-0432.CCR-15-2314.

[10]Shah, Sejal Siddharth, et al. “Genetic Alterations of the PIK3CA Oncogene in Human Oral Squamous Cell Carcinoma in an Indian Population.” Oral Surgery, Oral Medicine, Oral Pathology and Oral Radiology, vol. 120, no. 5, Elsevier Ltd, Nov. 2015, pp. 628–35, doi:10.1016/j.oooo.2015.08.003.

[11]Chen, Shu-Jen, et al. “Ultra-Deep Targeted Sequencing of Advanced Oral Squamous Cell Carcinoma Identifies a Mutation-Based Prognostic Gene Signature.” Oncotarget, vol. 6, no. 20, July 2015, pp. 18066–80, doi:10.18632/oncotarget.3768.

[12]Cortelazzi, Barbara, et al. “Receptor Tyrosine Kinase Profiles and Human Papillomavirus Status in Oropharyngeal Squamous Cell Carcinoma.” Journal of Oral Pathology and Medicine, vol. 44, no. 9, Oct. 2015, pp. 734–45, doi:10.1111/jop.12301.

[13]Anthony C. Nichols, David A. Palma, Winsion Chow, Susan Tan, Chandheeb Rajakumar, Giananthony Rizzo, Kevin Fung, Keith Kwan, Brett Wehrli, Eric Winquist, et al. “High Frequency of Activating PIK3CA Mutations in Human Papillomavirus–Positive Oropharyngeal Cancer.” JAMA Otolaryngology–Head & Neck Surgery, vol. 139, no. 6, June 2013, pp. 617–22, doi:10.1001/jamaoto.2013.3210.

[14]Cohen, Yoram, et al. “Mutational Analysis of PTEN/PIK3CA/AKT Pathway in Oral Squamous Cell Carcinoma.” Oral Oncology, vol. 47, no. 10, Elsevier Ltd, 2011, pp. 946–50, doi:10.1016/j.oraloncology.2011.07.013.

[15]Bruckman, Karl C., et al. “Mutational Analyses of the BRAF, KRAS, and PIK3CA Genes in Oral Squamous Cell Carcinoma.” Oral Surgery, Oral Medicine, Oral Pathology, Oral

Radiology, and Endodontology, vol. 110, no. 5, 2010, pp. 632–37, doi:https://doi.org/10.1016/j.tripleo.2010.05.002.

[16]Qiu, Wanglong, et al. “Novel Mutant-Enriched Sequencing Identified High Frequency of PIK3CA Mutations in Pharyngeal Cancer.” International Journal of Cancer, vol. 122, no. 5, Mar. 2008, pp. 1189–94, doi:10.1002/ijc.23217.

[17]Qiu, Wanglong, et al. “PIK3CA Mutations in Head and Neck Squamous Cell Carcinoma.” Clinical Cancer Research, vol. 12, no. 5, Mar. 2006, pp. 1441–46, doi:10.1158/1078-0432.CCR-05-2173.
