# Supplementary material for: PIK3CA Gene Mutations in HNSCC: Systematic Review and Correlations with HPV Status and Patient Survival
Source: Cancers (Basel). 2022 Mar 2;14(5):1286. doi: 10.3390/cancers14051286 (PMC8909011; doi:10.3390/cancers14051286)
Supplement: Supplementary file 1 [file cancers-14-01286-s001.zip › Table S1-Systematic review HNSCC PIK3CA qPCR.pdf]

		[1]	[2]	[3]	[4]	[5]	[6]	
		EM. Borkowska	García-	SB Pattle	JA. Theurer	SM. McBride	T Suda	Present Study
		2021	2018	2017	2015	2014	2012	2021
		Poland	Spain	Scotland	Indian	USA	Japan	Portugal
		113	62	100	25	64	115	95
**Samples specimens**		FFPE	biopsy Tumor	FFPE	biopsy Tumor	biopsy Tumor	biopsy Tumor	biopsy Tumor
**time collected**		surgery	surgery	non	untreated	treated	surgical	diagnostic
**demographics**								
gender	Male	86 (76%)	34 (55%)	63 (63%)	19 (76%)	21 (91%)	93 (81%)	70 (74%)
	Female	27 (24%)	28 (45%)	37 (37%)	6 (24%)	2 (9%)	22 (19%)	25 (26%)
range_ age		42–91	33-90	non	<60y n=7	non	non	
mean_age		63	68	66	≥60y n=18	non	65	62
**risk factors**								
consumptio	tobacco	84 (74%)	38 (61%)	47 (47%)	24 (96%)	13/23 (56.5%)	115 (100%)	**66 (70%)**
	alcohool	62 (55%)	non	76 (76%)	non	non	non	
**clinical parameters**								
histologic diagnostic		HNSCC	HNSCC	HNSCC	HNSCC	HNSCC	HNSCC	HNSCC
tumour location subsite	oral cavity	35 (31%)	non	0	11 (44%)	20 (31%)	31 (27%)	20 (21%)
	oropharynx	30 (26%)	non	52 (52%)	8 (32%)	23 (36%)	25 (22%)	75 (79%)
	larynx	48 (43%)	non	0	2 (8%)	8 (12%)	23 (20%)	0
	Hypophary	0	non	48 (48%)	2 (8%)	5 (8%)	25 (22%)	0
	Nasopharyn	0	non	0	2 (8%)	3 (5%)	11 (10%)	**0**
tumour stage	stage I	55 (49%)	non		3 (12%)	non	12 (10%)	8 (8%)
	stage II		non	57 (57%)	2 (8%)	non	23 (20%)	19 (20%)
	stage III	58 (51%)	non		2 (8%)	non	26 (23%)	26 (27%)
	stage IV		non	43 (43%)	18 (72%)	non	54 (47%)	40 (42%)
HPV status	detected	26 (23%)	0	28 (28%)	non	non	non	27(28%)
	not detected	87 (77%)	62 (100%)	70 (70%)	non	non	non	68 (72%)
p16(+)	detected	11 (10%)	non	26 (26%)	8 (32%)	non	non	36 (38%)
**Evaluation PIK3CA gene**								
PIK3CA-assay		qPCR	qPCR	qPCR	qPCR	qPCR	qPCR	RT-qPCR
overall Frequency PIK3CA mutation		9 (8%)	11 (18%)	17 (17%)	4 (16 %)	10 (16%)	37 (32%)	37* (39%)
copynumber/expression		non	non	yes	non	no	yes	no
frequency PIK3CA per mutation type — exon 9	E542K	non	evaluated	non	1	2	evaluated	1
	E545K	non	evaluated	non	1	6	evaluated	2
	E545D	non	evaluated	non	1	1	evaluated	27
exon 20	H1047R	9	evaluated	non	1	1	evaluated	1
	H1047L	non	non	non	non	0	evaluated	0
frequency PIK3CA per HN subsite	oral cavity	3	non	non	2	1	9	8
	oropharynx	0	non	14	2	6	10	29
	larynx	6	non	non	0	1	8	0
	Hypophary	non	non	3	0	0	9	0
	Nasopharyn	non	non	non	0	2	1	0
PIK3CA and HPV	mut	1	0	non	non	5	non	25
	mut	8	11	non	non	1	non	12
outcome								
OS		non	non	non	non	non	no association	no association
DFS		non	non	non	non	non	non	better
RFS		no correlation	non	non	non	non	non	non

Legend:

* 6 cases with 2 substitutions

non not described
