# Supplementary material for: PIK3CA Gene Mutations in HNSCC: Systematic Review and Correlations with HPV Status and Patient Survival
Source: Cancers (Basel). 2022 Mar 2;14(5):1286. doi: 10.3390/cancers14051286 (PMC8909011; doi:10.3390/cancers14051286)
Supplement: Supplementary file 1 [file cancers-14-01286-s001.zip › Table S2-Overall 17 articles selected for HNSCC PIK3CA mutation.pdf]

		[1]	[2]	[3]	[4]	[5]	[6]	[7]	[8]	[9]	[10]	[11]	[12]	[13]	[14]	[15]	[16]	[17]
		Weiping Jie	Abeer A.	V. Denninghoffa	Kulsoom Ghias	Sunny Haft	Sun Min Lim	D.	Ali M. Al-Amri	Nicole G. Chau	Sejal Shah	Shu-Jen Chen	Barbara	Nichols AC	Yoram Cohen	Karl C.	Qiu W	Qiu W
		2021	2020	2020	2019	2019	2019	2016	2016	2016	2015	2015	2015	2013	2011	2010	2008	2006
		China	Saudi Arabia	Argentina	Pakistan	USA	Korea	USA	Saudi Arabia	USA	India	Taiwan	Italy	USA	Israel	USA	USA	USA
		26	44	49	7	46	92/93	75/93	48	213	50	345	76	87	37	35	24	30
**Samples specimens**		tumour tissue	tumour tissue	tumour tissue	tumour tissue	tumour tissue	tumour tissue	tumour tissue	tumour tissue	tumour tissue	tumour tissue	tumour tissue	tumour tissue	tumour tissue	tumour tissue	tumour tissue	tumour tissue	tumour tissue
**time collected**		surgery	diagnostic	non	treatment-naïve	non	treatment-naïve	surgery	treatment-naïve	non	surgery	treatment-naïve	surgery	treatment-naïve	non	diagnostic	surgery	non
**demographics**																		
gender	Male	20	23 (52%)	36 (49%)	5	41 (89%)	75 (81%)	90	32	158	36 (72%)	325 (94%)	non	71	16	19	19	non
	Female	6	21 (48%)	38 (51%)	2	5 (11%)	18 (19%)	3	16	55	14 (28%)	20 (6%)	non	16	21	16	5	non
range_ age		35-79	21-93	41-93	35-71	35-74	28-80	36-86	18-90	22-88	27-70	27-89	non	<60y (n=51)	24-90	28-91	38-78	40-85
mean_age		61	56	68	54	56	59	60	54	59	47	50	non	≥60y (n=36)	70	69	58	64
**clinical parameters**																		
histologic diagnostic		SCC	SCC	SCC	SCC	SCC	SCC	SCC	SCC	SCC	SCC	SCC	SCC	SCC	SCC	SCC	SCC	SCC
tumour location subsite	oral cavity	11 (42.3%)	44	47	6	0	35 (38%)	0	non	60	50	345	0	0	37	45	0	8
	oropharynx	6 (23.1%)	0	2	0	46	26 (28%)	0	non	97	0	0	76	87	0	0	0	0
	larynx	0	0	0	0	0	12 (13%)	41	14 (29%)	24	0	0	0	0	0	0	0	10
	Hypopharynx	0	0	0	0	0	15 (16%)	34	9 (19%)	6	0	0	0	0	0	0	24	6
	Nasopharynx	0	0	0	1	0	5 (5%)	0	20 (42%)	12	0	0	0	0	0	0	0	2
	not stratified	0	0	0	0	0	0	0	5 (10%)	14	0	0	0	0	0	0	0	4
HPV status	detected	2 (8%)	10 (23%)	non	0	46	20 (22%)	0	non	103 (48%)	non	non	17 (22%)	46 (53%)	non	non	non	non
	not detected	24 (92%)	34 (77%)	non	7	0	56 (60%)	75	non	34 (16%)	non	non	59 (78%)	41 (47%)	non	non	non	non
**Evaluation PIK3CA gene**																		
PIK3CA-assay		SANGER	NGS	SANGER	WES	WES	NGS	NGS	SANGER	NGS	SANGER	NGS	SANGER	SANGER	SANGER	SANGER	SANGER	SANGER
assay target analysis		DNA	DNA	DNA	DNA	DNA	DNA	DNA	DNA	DNA	DNA	DNA	DNA	DNA	DNA	DNA	DNA	DNA
Overall Frequency PIK3CA mutation		8 (31%)	16 (36%)	16 (32%)	1 (14%)	13 (28%)	19/92 (21%)	10/75 (13%)	14 (29%)	28 (13%)	18 (36%)	58 (17%)	2 (3%)	17 (20%)	4 (11%)	1 (3%)	5 (21%)	4 (11%)
frequency PIK3CA per exon hotspot mutation	exon 9 (*)	detected	13	8	1	9	5	1	7	detected	2	7	2	13	2	1	3	2
	exon 20 (#)	1	3	8	0	1	non	9	2	detected	15	45	non	4	2	0	2	2
	not stratified/novel (+)	18	non	non	non	3	14	non	5	non	1	6	non	non	non	0	0	8
frequency PIK3CA per HN subsite	oral cavity	non	16	16	1	non	non	0	non	non	18	58	non	non	4	1	non	1
	oropharynx	non	non	non	non	non	non	0	non	non	non	non	2	17	non	non	non	0
	larynx	non	non	non	non	non	non	8	non	non	non	non	non	non	non	non	non	0
	Hypopharynx	non	non	non	non	non	non	2	8	non	non	non	non	non	non	non	5	3
	Nasopharynx	non	non	non	non	non	non	0	non	non	non	non	non	non	non	non	non	0
PIK3CA and HPV	mut PIK3CA HPV(+)	non	non	non	0	13	4	0	non	non	non	non	1	13	non	non	non	non
	mut PIK3CA HPV(-)	non	non	non	1	non	3	10	non	non	non	non	1	4	non	non	non	non
HNSCC PIK3CA mutation outcome	OS	non	better (p=0.09)	non	non	non	better (p=0,0755)	non	non	not associated	non	non	non	non	better (p=0.761)	non	non	non
	DFS	non	non	non	non	non	non	better (p=0.063)	non	non	non	non	non	non	non	non	non	non
	RFS	non	non	non	non	non	non	non	non	non	non	non	non	non	non	non	non	non
**PIK3CA mutation profile**																		
		A533T<br> C535P<br>* E522V<br>* T5441I<br># A990V<br># D1056G<br># F998S<br># F1016L<br># H994Y<br># H1047R<br># H1065R<br># I1062T<br># S1008P<br># S1015S<br># T1061A<br># Q981<br># Q1033<br># Q1064<br># W1057	* E545K<br># p.A1066T	* E542 K (n=1)<br>* E545 K (n=6)<br>* F550L (n=1)<br>* E545 G<br>* E545Q<br>* Q546L<br># M1043I (n=8)<br># M1043V<br># H1047L<br># H1047R	* E545 K (n=1)	* E542 K (n=1)<br>* E545 K (n=8)<br># G1049R (n=1)	* E545 K (n=5)	* E542 K (n=1)<br>* E545 K<br># H1047R (n=9)	* E542K (n=7)<br># T1025T (n=2)<br>+ * S541S (n=1)<br>+ * R516K (n=1)<br>+ # T1053A (n=1)<br>+ # Y1021Y (n=1)<br>+ # N1068S (n=1)	* E542K<br>* E545K<br># H1047R	* E542K (n=1)<br>* E545K (n=1)<br># A994A (n=1)<br># T1025T (n=14)<br>+ C2985T (n=1)	* D549<br>* E542<br>* E545<br># A1046<br># G1049<br># H1047R<br># H1048<br># M1043	* E542K<br>* E545K	* E542K (n=8)<br>* E545K (n=5)<br># H1047R (n=4)	* E542K<br>* E545K (n=2)<br># G1049R (n=1)<br># H1047R (n=1)<br># H1047L<br># H1047Y	* E545K (n=1)	* E542K (n=1)<br>* E545K (n=1)<br>* E542G (n=1)<br># M1040V (n=1)<br># H1047R (n=1)	* E542K (n=1)<br>* E545K (n=1)<br># H1047R (n=1)<br>+ Y343C (n=1)
