# Supplementary material for: PIK3CA Gene Mutations in HNSCC: Systematic Review and Correlations with HPV Status and Patient Survival
Source: Cancers (Basel). 2022 Mar 2;14(5):1286. doi: 10.3390/cancers14051286 (PMC8909011; doi:10.3390/cancers14051286)
Supplement: Supplementary file 1 [file cancers-14-01286-s001.zip › Table S3-HNSCC PIK3CA Mutational profile.pdf]

serie number	mutation type_cat	studies reported	*n* cases	exon
1	A990V	1	qualitative	20
2	A1046	1	qualitative	20
3	A533T	1	qualitative	9
4	A994A	1	1	20
5	C2985T	1	1	9
6	C535P	1	qualitative	9
7	D1056G	1	qualitative	20
8	D549	1	qualitative	9
9	E542G	1	1	9
10	E542K	9	21	9
11	E522V	1	qualitative	9
12	E545K	15	31	9
13	E545G	1	qualitative	9
14	E545Q	1	qualitative	9
15	F1016L	1	qualitative	20
16	F550L	1	1	9
17	F998S	1	qualitative	20
18	G1049	3	2	20
19	H1047L	2	qualitative	20
20	H1047R	9	16	20
21	H1047Y	1	qualitative	20
22	H1048	1	qualitative	20
23	H1065R	1	qualitative	20
24	H994Y	1	qualitative	20
25	I1062T	1	qualitative	20
26	M1040V	1	1	20
27	M1043I	2	8	20
28	M1043V	1	qualitative	20
29	N1068S	1	1	20
30	p.A1066T	0	qualitative	20
31	Q1033	0	qualitative	20
32	Q1064	0	qualitative	20
33	Q546L	0	qualitative	9
34	Q981	0	qualitative	20
35	R516K	1	1	9
36	S1008P	0	qualitative	20
37	S1015S	0	qualitative	20
38	S541S	1	1	9
39	T1025T	2	16	20
40	T1053A	1	1	20
41	T1061A	0	qualitative	20
42	T5441l	0	qualitative	9
43	W1057	0	qualitative	20
44	Y1021Y	1	1	20
45	Y343C	1	1	no data
