# Supplementary material for: PIK3CA Gene Mutations in HNSCC: Systematic Review and Correlations with HPV Status and Patient Survival
Source: Cancers (Basel). 2022 Mar 2;14(5):1286. doi: 10.3390/cancers14051286 (PMC8909011; doi:10.3390/cancers14051286)
Supplement: Supplementary file 1 [file cancers-14-01286-s001.zip › Table S4-Clinical and demographic characterization of the 295 excluded cases.pdf]

Table S4: Clinical and demographic characterization of the 295 excluded cases

Variables description		*n*	%
Gender	Male	227	76.9%
	Female	68	23.1%
Age	<65	195	66.1%
	≥65 Years	100	33.9%
Consumptions	Active	222	75.3%
	No	40	13.6%
	No data	33	11.2%
Tumour Local	Oropharynx	127	43.1%
	Oral Cavity	168	56.9%
TNM stage	I	107	36.3%
	II	90	30.5%
	III	79	26.8%
	IV	2	0.7%
	No data	17	5.8%
p16 marker	p16 INK4a (-)	5	1.7%
	p16 INK4a (+)	2	0.7%
	No data	288	97.6%
HPV status	HPV(-)	210	79.2%
	HPV(+)	85	28.8%
Treatment	surgery	39	13.2%
	Radiation	29	9.8%
	Combine Medicine	212	71.9%
	No data	15	5.1%
Treatment response	Complete	195	66.1%
	Persistence	29	9.8%
	Relapse	24	8.1%
	no data	47	15.9%
