# Supplementary figures and images for: PIK3CA Gene Mutations in HNSCC: Systematic Review and Correlations with HPV Status and Patient Survival
Source: Cancers (Basel). 2022 Mar 2;14(5):1286. doi: 10.3390/cancers14051286 (PMC8909011; doi:10.3390/cancers14051286)

Figure S1: Participant flow chart

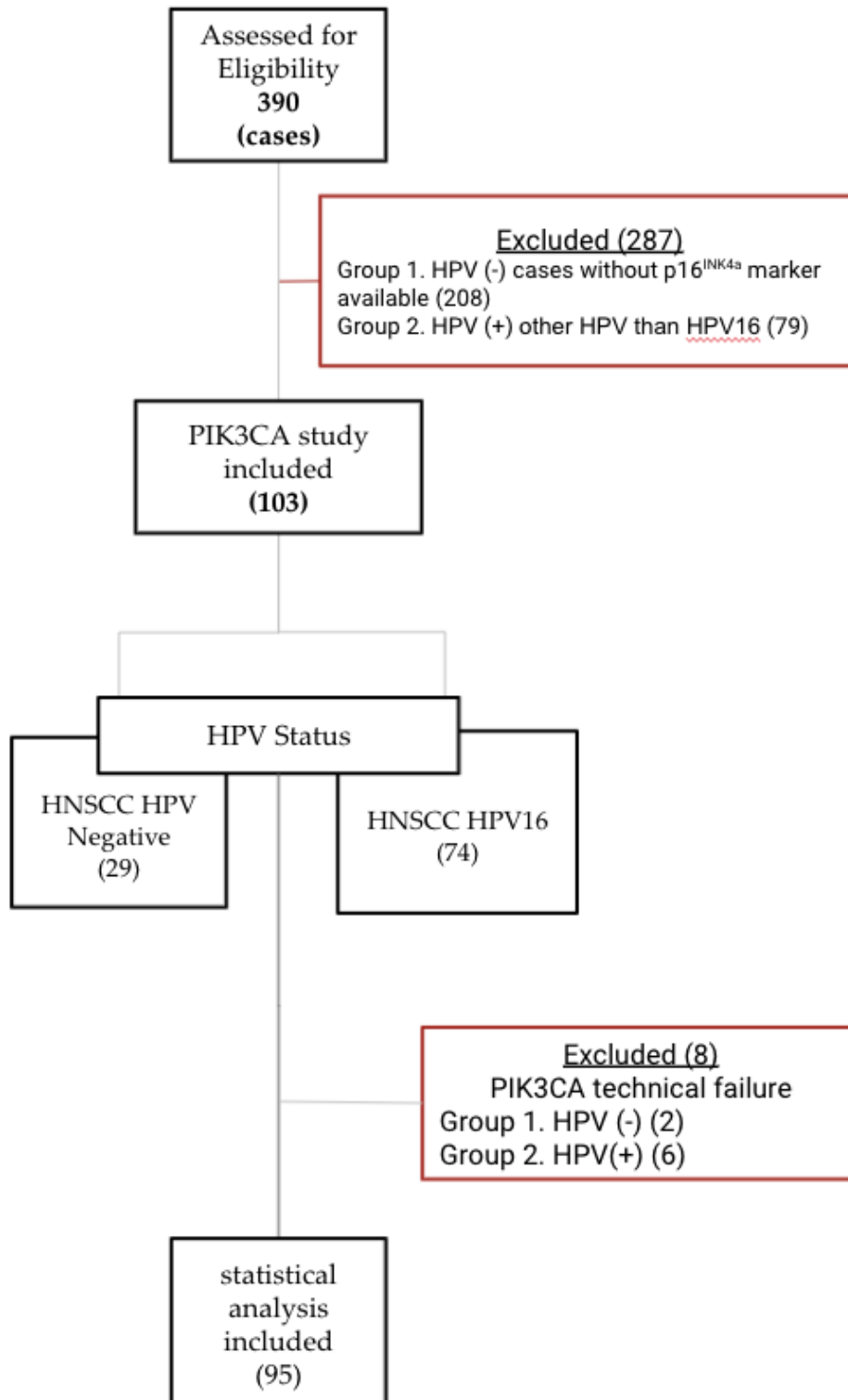

Supplement: Supplementary file 1 [file cancers-14-01286-s001.zip › Figure S1-Participant flow chart.pdf]

Figure S2:Kaplan-Meier overall survival curves for the included and excluded groups

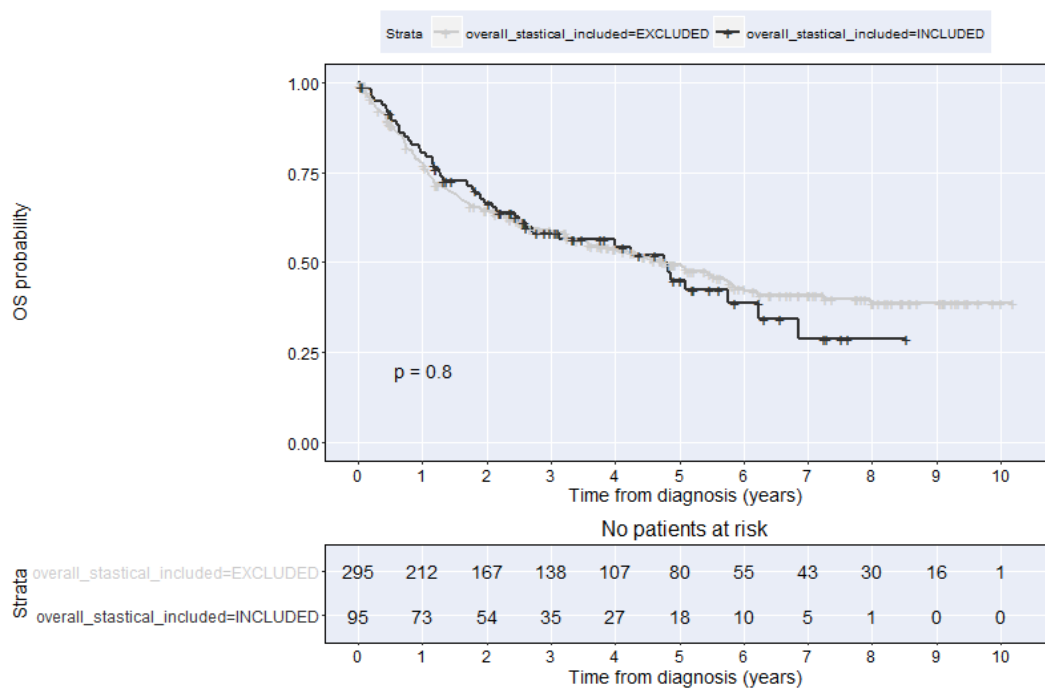

Supplement: Supplementary file 1 [file cancers-14-01286-s001.zip › Figure S2-Kaplan-Meier overall survival curves for the included and excluded groups.pdf]
